# Comparison of the effects of using feedback devices for training or simulated cardiopulmonary arrest

**DOI:** 10.1186/s13019-024-02669-z

**Published:** 2024-03-27

**Authors:** Jinghao Jiang, Jinghuang Yan, Dongai Yao, Jinsong Xiao, Rongtao Chen, Yan Zhao, Xiaoqing Jin

**Affiliations:** 1 Emergency Center, Zhongnan Hospital of Wuhan University, 169 Donghu Road, Wuchang, Wuhan, Hubei 430071 China; 2 Emergency department, The First People’s Hospital of Wuxue, Wuxue, Hubei 435400 China; 3 Physical Examination Center, Zhongnan Hospital of Wuhan University, Wuhan, Hubei 430071 China; 4 Department of Neurology, Zhongnan Hospital of Wuhan University, Wuhan, Hubei 430071 China; 5 Emergency department, The Second People’s Hospital of China Three Gorges University, No. 21, Xiling road, Xiling district, Yichang, Hubei 443000 China

**Keywords:** Cardiopulmonary resuscitation, Audiovisual feedback, Training, Simulated cardiopulmonary arrest, Chest compression

## Abstract

**Background:**

High-quality chest compression is essential for successful cardiac arrest resuscitation. High-quality cardiopulmonary resuscitation (CPR) can effectively improve the survival rate of patients with cardiopulmonary arrest. However, bystanders untrained in cardiopulmonary resuscitation may provide inadequate chest compressions. Previous studies have shown that the use of feedback devices in training alone or in simulated cardiopulmonary arrest alone can improve cardiopulmonary resuscitation. This study aims to determine whether using an audiovisual feedback (AVF) device during CPR training or a simulated cardiopulmonary arrest (CA) scenario would be more effective in improving the quality of chest compressions (CC).

**Methods:**

We use a prospective, randomized, 2 × 2 factorial design trial. A total of 160 participants from Wuhan University and senior clinical medicine undergraduates who had not participated in any CPR training before and had no actual CPR experience are recruited. Each participant is randomized to 1 of 4 permutations, including AVF device vs. no AVF device during CPR training and AVF device vs. no AVF device during simulated CA. Main outcomes and measures are the depth, the percentage of CCs with correct depth (5–6 cm), the rate of CCs, and the percentage of CCs with the correct rate (100–120 cpm).

**Results:**

The use of the AVF device during simulated CA resulted in improved CC quality. In CA without AVF device, the average compression depth and the percentage of adequate depth with AVF device are 5.1 cm, 5.0 cm and 55.5%, 56.3%, respectively, which are higher than those without AVF device (4.5 cm, 4.7 cm and 32.8%, 33.6%). (*p* = 0.011, *p* = 0.000, both < 0.05).Compared with CA without AVF device, the average compression rate and the percentage of adequate rate with AVF device are 112.3 cpm, 111.2 cpm and 79.4%, 83.1%, respectively. The average compression rate and the percentage of adequate rate without using the AVF device are 112.4 cpm, 110.3 cpm and 71.5%, 68.5%, respectively. (*p* = 0.567 > 0.05, *p* = 0.017 < 0.05)Although the average compression rate in group D is slightly lower than that in group C, the percentage of suitable frequency with the feedback device is still higher than that without AVF device.

**Conclusion:**

Using a feedback device during simulated cardiopulmonary arrest is more effective in improving cardiopulmonary resuscitation than during training.

**Supplementary Information:**

The online version contains supplementary material available at 10.1186/s13019-024-02669-z.

## Introduction

In many cases, cardiac arrest occurs suddenly, and quick and effective treatment is urgently needed [1]. Out-of-hospital cardiac arrest (OHCA) is a worldwide health issue that has received much attention [2]. In Asian countries, OHCA has become an increasingly significant concern and cause of death [3]. Its incidence is 41.8/100 000 among the general population in China [4]. According to statistics, the survival rate after adult OHCA is only about 7% on average [5]. About 47% of OHCAs are witnessed by bystanders [6]. However, many of them do not know how to respond to such cases.

The 2020 American Heart Association (AHA) guidelines highlight the first line of the OHCA Chain of Survival as recognition of cardiopulmonary arrest (CA) by bystanders and that the bystander’s reactions and skills of cardiopulmonary resuscitation (CPR) determine the survival of patients [7]. Although advanced life support (ALS) interventions are commonly believed to increase OHCA survival, high-quality bystander CPR is still a fundamental strategy for improved survival [8, 9]. High-quality CPR is characterized by a compression rate between 100 and 120 compressions per minute (CPM), a compression depth between 5 and 6 cm, allowing complete chest recoil after each compression, minimizing interruptions in chest compressions (CC), and avoiding excessive ventilation. Currently, there is research on applying audiovisual feedback (AVF) devices to CPR, aiming to improve CPR quality [10, 11]. Recent evidence has also shown that using feedback devices during actual cardiac arrest can improve the quality of CPR [12, 13]. However, the current dilemma is that feedback devices are not always available at training sites or medical institutions.

Therefore, this prospective randomized study aims to investigate whether using an AVF device in CPR training or in a simulated CA scenario is more conducive to improving the quality of CC.

## Methods

### Participants and setting

We obtained approval from the Ethics Committee of Zhongnan Hospital of Wuhan University, Hubei Province, China. We also obtained written informed consent from participants and informed all participants that we would evaluate their performance for scientific purposes only. All procedures are performed according to the Declaration of Helsinki. The research method used in the experiment is a cross-sectional study and is conducted at the Clinical Skill Training Center of the Second Clinical Medical College of Wuhan University over one weekend. All participants were fourth-year undergraduate students specializing in Clinical Medicine. Notably, 160 participants with no previous CPR training and no actual CPR experience are randomly assigned to 4 groups.

Upon inclusion in this study, all students received CPR training on a manikin following a standardized teaching protocol of the medical school, according to the ILCOR guideline for adult basic life support. Furthermore, the height and weight of all participants are recorded.

### Study design

We used a prospective, randomized, 2 × 2 factorial design trial with an explicit method [14]. The instructor was an AHA BLS instructor, and he explain the correct method of CPR. All participants were randomly divided into four groups: Group A (without an AVF device during CPR training and simulated CA) (T-S-); Group B (without an AVF device during CPR training and with an AVF device during simulated CA) (T-S+); Group C (with an AVF device during CPR training and without an AVF device during simulated CA) (T + S-); and Group D (with an AVF device during CPR training and simulated CA) (T + S+). The study consist of three phases: First, all participants watched a 30-minute video of CPR, and the instructor emphasize all points of CPR. Then, they practice on the adult Resusci Anne QCPR manikin (Laerdal China Ltd., Hangzhou, China) during CPR training with or without the AVF device (Group C and Group D receive the AVF device, Group A and Group B not) until they passed the assessment. Each group was guided by an AHA trainer who had no knowledge of our experimental grouping. All participants practice at least 3 times, 2 min each time. Finally, they took part in a 2-minute continuous CC during simulated CA with or without the AVF device (Group B and Group D received the AVF device, Group A and Group C not). The rate and depth of CCs were recorded, and technical performance data were recorded by the Laerdal Computerized Skill Reporting System and analyzed Later. All participants were divided into 4 groups by random number table method. Due to the technical problems of data acquisition, 23 participants were excluded, resulting in a total of 137 participants. Therefore, groups A, B, C, and D had 31, 38, 36, and 32 students, respectively.

**Fig. 1:**
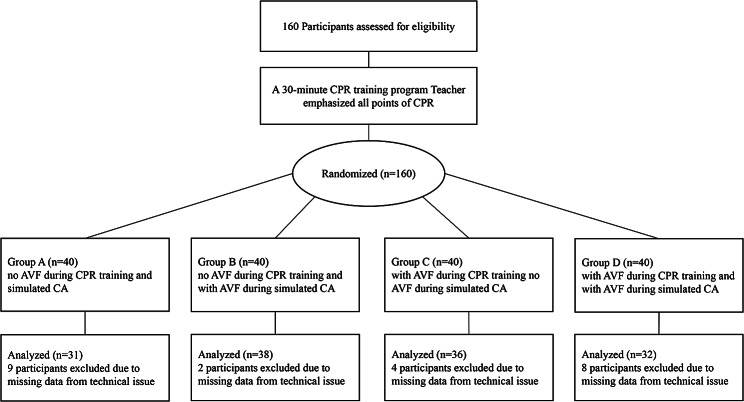
Flow chart of design and recruitment of participants

For this study, we use SimPad PLUS as the AVF equipment in this study. The AVF device is a tablet that can give us timely feedback on depth and rate of CCs to adjust the compression to improve our compression quality. The Laerdal SimPad PLUS is a device for simulating cardiopulmonary resuscitation (CPR) training and is an emulation controller that can be used with an emulation human. Specifically, SimPad PLUS can control and record different parameters of the model, including compression depth, compression frequency, ventilation and other parameters, so as to simulate different clinical situations and improve the realism and effect of first aid training. The study protocol is summarized in Fig. 1.

### Measurements

We analyzed the depth and rate of CCs, the percentage of CCs with correct depth (5–6 cm), the rate of CCs, and the percentage of CCs with the correct rate (100–120 cpm) among groups according to with or without feedback devices during CPR training or simulated CA scenarios.

### Sample size calculation

We determine the required group sample size by power analysis (G*Power 3.1.9.7). From these calculations, four groups required at least 82 participants in total sample size to have a statistical power of 80% and a type I error of 0.05 to detect the estimated difference.

### Data analyses

All data were analyzed with SPSS software (SPSS version23; IBM Corporation). The study used multivariate linear regression analysis. Comparisons between multiple groups are performed using analysis of variance (ANOVA) and least significant difference (LSD) correction for the post-hoc comparison. Data are expressed as numbers, percentages or means with their 95% CIs. *P* < 0.05 was considered statistically significant.

## Results

### Demographic characteristics of the participants

The demographics of the four groups are summarized Table 1. There were no significant differences noted in height or weight between groups.

**Table 1:** Characteristics among the four groups. (Mean, 95% CI)

95%CI	Group A (T-S-)	Group B (T-S+)	Group C (T + S-)	Group D (T + S+)	ANOVA (P)
Height (cm)	164.3 (161.3-167.3)	167.0 (164.8-169.2)	164.7 (162.2-167.1)	166.8 (164.4-169.1)	0.268
Weight (kg)	55.1 (51.7–58.5)	55.8 (53.8–57.9)	55.2 (51.8–58.5)	55.1 (52.9–57.4)	0.975
BMI (kg/m^2^)	20.3 (19.5–21.1)	20.0 (19.5–20.5)	20.2 (19.5–21.0)	19.8 (19.3–20.3)	0.663
Men vs. Women	1.1	1	1	1	NA

BMI: Body Mass Index

ANOVA: Analysis of the generated main effect p-values

T-: no AVF during CPR training

T+: with AVF during CPR training

S-: no AVF during simulated cardiopulmonary arrest

S+: with AVF during simulated cardiopulmonary arrest

### The effect of the AVF device on the quality of CC during training and simulated CA

The mean percentage of CCs with adequate depth (5–6 cm) in the four groups and the percentage of CCs with an adequate rate (100–120 cpm) are shown in Table 2 and the measurements encompass both the training and simulated CA phases.

**Table 2:** Chest compression quality of four groups (mean, 95%CI)

95%CI	Group A (T-S-)	Group B (T-S+)	Group C (T + S-)	Group D (T + S+)	ANOVA (P)
CC depth (cm)	4.7 (4.4–5.1)	5.0 (4.9–5.2)	4.5 (4.2–4.8)	5.1 (5.0-5.3)	0.011
Adequate depth (%)	33.6 (21.7–45.6)	56.3 (46.5–66.1)	32.8 (22.8–42.9)	55.5 (49.0-62.1)	0.000
CC rate (cpm)	110.3 (106.9-113.7)	111.2 (109.7-112.7)	112.4 (109.7–115.0)	112.3 (110.4-114.1)	0.567
Adequate rate (%)	68.5 (59.6–77.4)	83.1 (78.1–88.1)	71.5 (62.4–80.7)	79.4 (73.4–85.3)	0.017

CC: Chest Compression

ANOVA: Analysis of the generated main effect p-values

T-: no AVF during CPR training

T+: with AVF during CPR training

S-: no AVF during simulated cardiopulmonary arrest

S+: with AVF during simulated cardiopulmonary arrest.

**Fig. 2:**
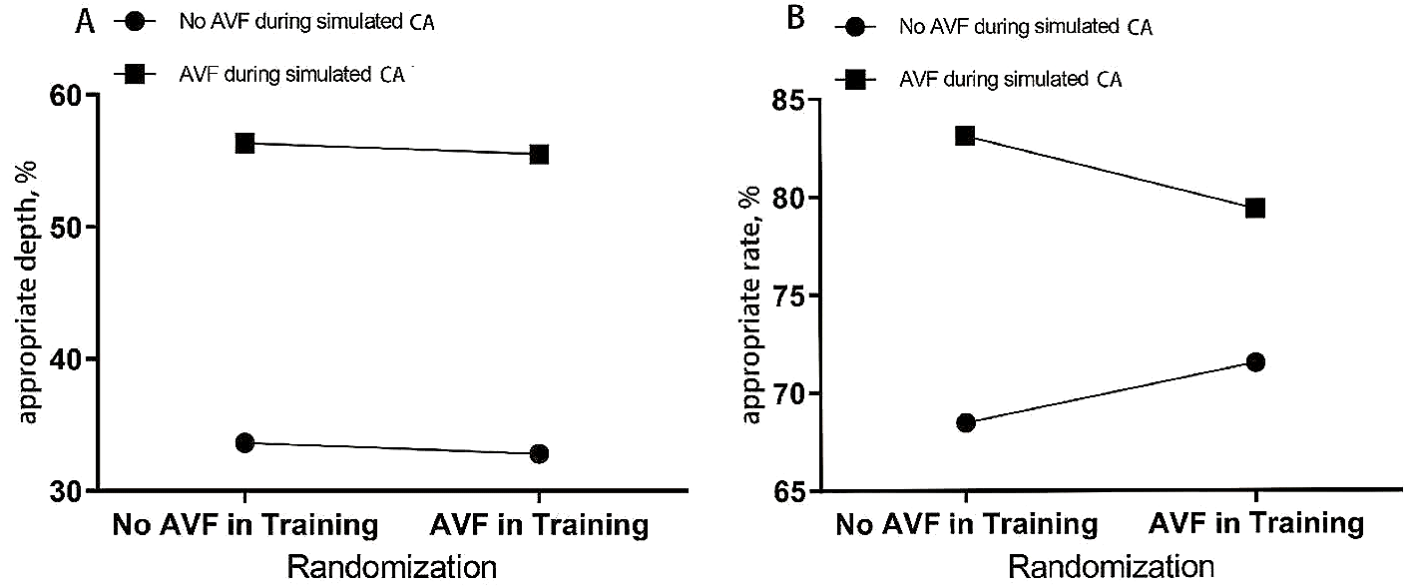
Effects of the AVF device used in training and simulated CA on CC Quality. (**A**) The mean proportion of CCs with appropriate depth between 5 and 6 cm. (**B**) The mean proportion of CCs with a rate of 100–120 per minute. AVF: audiovisual feedback. CA: simulated cardiopulmonary arrest. CC: chest compression

Figure 2 shows the main effect of the AVF device during CPR training and simulated CA on the quality of CCs.

## Discussion

This study aims to evaluate whether using an AVF device during CPR training or a simulated CA scenario is more conducive to improving the quality of CC. Our study is similar to and differs from previous studies that have examined the effect of feedback devices on the quality of CPR [14]. First, the two studies use different feedback devices. Second, the two studies are conducted in different settings and different populations. Our study was conducted on a university campus, while the Cheng et al. study was conducted in an urban pediatric emergency department. Also, our study population and Cheng’s study population were different. Our study included fourth-year medical students with no formal CPR training, and they are not professional medical personnel, whereas the study population of Cheng et al. included professional healthcare personnel with CPR training [14]. Notably, our results indicate that using the AVF device during simulated CA can improve the quality of CCs. Typically, when performing CPR, there is often a lack of an AVF device to assess the depth of CCs. Moreover, as the duration of CPR increases, participants may experience fatigue, making it challenging to achieve the appropriate CC depth and rate [15]. Importantly, Zhou et al. indicate that using feedback devices helps improve the quality of CPR [16], other authors shared this opinion [12, 17, 18].

Our study expands upon previous observations regarding the effect of AVF devices on the quality of chest compressions (CC) during CPR training and simulated cardiac arrest scenarios. Our findings have practical implications for recommending the use of more feedback devices in public places such as hospitals and other public locations. During a simulated CA scenario, the adequate depth percentage (55.5%, 56.3%) and the adequate rate percentage (79.4%, 83.1%) of the group using AVF devices were higher than those of the group without AVF devices (32.8%, 33.6%), (71.5%, 68.5%).This means using an AVF device has significantly improved the percentage of CCs with appropriate depth and rate. It’s worth noting that the rate of CCs in all groups met the 2020 AHA Guidelines for recommendations [7], with a rate of 100–120 compressions per minute. Although the rate of CCs varied between groups, they were all within the appropriate range without statistical differences. Consistent rates can be achieved through practicing with songs of a specific beat, which can help participants maintain a stable rhythm [19]. During a simulated CA scenario, the AVF device improve the CC quality by increasing the proportion of appropriate rate and depth, and the AVF device’s application significantly improved the CC quality. These results are in line with those of a previous study, which has already shown that the application of the AVF device is effective for refreshing CPR skills in a simulated cardiac arrest scene [14]. There may be several reasons for the poor performance of feedback devices in training: (1) The people we tested are all college students who had not received CPR training before, and the training time is too short to form physical memory; (2) For novices, using a feedback device during training and not using a feedback device during simulated CA may lead to psychological dependence on the feedback device during training and excessive attention to the feedback device data, resulting in poor CPR quality during simulated CA due to lack of feedback. Therefore, the use of the AVF device during simulated CA scenarios is more conducive to improving the quality of CC. But we still hoped using feedback device during training but not testing would have a sustained effect on the quality of CPR as often these devices are not available at the scene. Further controlled trials in different populations are needed to validate our results, such as separate trials involving bystanders and CPR-certified health professionals, or training prolonged enough for somatic memory to form.

### Limitations

Several factors may influence the effectiveness of CPR, including how well rescuers respond to visual feedback devices, patient age, and the duration of training. Additionally, the performance of the device may play a role in these outcomes. Our study aims to evaluate individual models under ideal laboratory conditions, but it is essential to consider that performing CPR in clinical practice can be more challenging, particularly with various patients [20]. Hence, further study is needed to apply a real-time feedback device in resuscitation from sudden cardiac arrest. Additionally, since we do not conduct follow-up surveys of the participants, we are unable to determine if their CPR quality differs from training or CC for a long time. As a result, further investigations into using a feedback device for regular assessment are required [21, 22]. One of the primary limitations of this study is that we use manikins for the experiment. Fourth-year undergraduates are senior clinical students and possess medical knowledge; therefore, they may not represent lay rescuers. In real-world clinical scenarios, it is recognized that cardiopulmonary resuscitation (CPR) may be administered by untrained bystanders. To enhance the CPR performance of untrained bystanders, future initiatives include the development of a user-friendly guidance and feedback system designed to be simple and comprehensible. This system aims to assist untrained bystanders in promptly executing correct CPR measures. Additionally, exploration is underway for wireless-connected portable CPR devices, enabling bystanders to respond to emergencies more effectively without the need for extensive professional training [23]. Another avenue involves the design of a simplified CPR apparatus, emphasizing ease of operation, ensuring it can be quickly utilized by bystanders even without formal training. Furthermore, there is an emphasis on widespread public awareness campaigns and training activities to augment public understanding of the significance of CPR. This multifaceted approach aims to boost confidence and capabilities among individuals who have not undergone specific CPR training, fostering a more competent response to emergency situations [24–26]. As mentioned in the introduction, the authors state that AVF device can improve the outcomes of OHCA. Furthermore, another limitation of our study is that we did not collect long-time training or chest compression test data on all participants’ CPR quality. We are unaware if using AVF devices can improve CPR quality over an extended period. Consequently, we plan to design a long-term training or chest compression test to estimate these participants’ CPR quality after using the AVF device during CPR training and a simulated CA scenario.

## Conclusions

The AVF device can improve the compression depth of CPR and the quality of chest compression when simulating CPR compared with CPR training when CPR training is performed first and then simulated CPR is performed. When defibrillators are provided in public places, providing feedback equipment at the same time can improve the quality of CPR for bystanders and likely improve survival outcomes.

### Electronic supplementary material

Below is the link to the electronic supplementary material.

Supplementary Material 1

## Data Availability

Not applicable.

## Abbreviations

CPR: Cardiopulmonary Resuscitation
AVF: Audiovisual Feedback
CA: Cardiopulmonary Arrest
CC: Chest Compression
OHCA: Out-of-Hospital Cardiac Arrest
AHA: American Heart Association
ALS: Advanced Life support
CPM: Compressions Per Min
LSD: least significant difference
